# Determination of the Solid Electrolyte Interphase Structure Grown on a Silicon Electrode Using a Fluoroethylene Carbonate Additive

**DOI:** 10.1038/s41598-017-06555-8

**Published:** 2017-07-24

**Authors:** Gabriel M. Veith, Mathieu Doucet, Robert L. Sacci, Bogdan Vacaliuc, J. Kevin Baldwin, James F. Browning

**Affiliations:** 1 Materials Science and Technology Division, Oak Ridge National Laboratory, Oak Ridge, TN 37831 USA; 2 Neutron Data Analysis and Visualization Division, Oak Ridge National Laboratory, Oak Ridge, TN 37831 USA; 3 Research Accelerator Division, Oak Ridge National Laboratory, Oak Ridge, TN 37831 USA; 4 Materials Science and Technology Division, Los Alamos National Laboratory, Los Alamos, NM 87544 USA; 5 Chemical and Engineering Materials Division, Oak Ridge National Laboratory, Oak Ridge, TN 37831 USA

## Abstract

In this work we explore how an electrolyte additive (fluorinated ethylene carbonate – FEC) mediates the thickness and composition of the solid electrolyte interphase formed over a silicon anode *in situ* as a function of state-of-charge and cycle. We show the FEC condenses on the surface at open circuit voltage then is reduced to C-O containing polymeric species around 0.9 V (vs. Li/Li^+^). The resulting film is about 50 Å thick. Upon lithiation the SEI thickens to 70 Å and becomes more organic-like. With delithiation the SEI thins by 13 Å and becomes more inorganic in nature, consistent with the formation of LiF. This thickening/thinning is reversible with cycling and shows the SEI is a dynamic structure. We compare the SEI chemistry and thickness to 280 Å thick SEI layers produced without FEC and provide a mechanism for SEI formation using FEC additives.

## Introduction

Of all the factors that limit battery performance (electrode design, ion transport, structural stability, etc) the most important, and least understood are the reactions that occur at the electrolyte-electrode interface^1^. At the anode the electrolyte (solvent and salt) is reduced forming a mixture of inorganic (e.g. LiF, POF) and organic/polymeric (e.g. C-O-C, Li-ROCO_2_) species which coat the surface^2–12^. This coating is referred to as the solid electrolyte interphase (SEI) that when formed correctly is self-terminating and effectively prevents further electrolyte reduction. Preventing further electrolyte reduction is important for safety and for maintaining battery life and performance as the Li in the SEI comes from the cathode. In the case of graphite, the SEI layer prevents the exfoliation of the graphite and prevents further reducing reactions, enabling long term cycling of the battery by maintaining the liquid electrolyte^1–12^.

Despite the importance of the SEI, we still do not know its structure (fraction polymeric/inorganic and their spatial orientation) or composition as a function of state-of-charge. More importantly we don’t know how these variables change as a function of cycling, voltage cut-off, or differ between balanced (matching cathode and anode capacities) versus unbalanced (usually excess Li) batteries^1^. This problem is particularly acute for the next generation of anode materials like silicon, which have higher theoretical capacity (Li_15_Si_4_ – 3579 mAh/g) compared to graphite (~330 mAh/g) and will enable lighter and lower volume anode configurations. In the case of silicon, the same electrolytes used for graphite anodes (e.g. 1.2 M LiPF_6_ - ethylene carbonate:dimethyl carbonate (EC/DMC) (3:7 wt%)) react with the silicon, forming a “bad” SEI layer. This “bad” SEI layer continues to react with the electrolyte, consuming it and limiting cycle life. One method to improve the SEI chemistry over silicon is the addition of chemical additives^13^. The most important, and widely used additive, is fluorinated ethylene carbonate (FEC). This material reduces at a higher potential (1.1–1.2 V) than other electrolyte components such as ethylene carbonate (0.7–0.8 V)^14^. This preferential reduction results in a better SEI layer that enables much longer cycling (hundreds of cycles) over a conventional electrolyte (tens of cycles) but the performance does not reach the level obtained for graphite^7, 8, 15^. In addition the SEI with FEC is more thermally stable (up to about 200 °C) than a traditional SEI (~153 °C)^16^. To date, there is no clear understanding of why the FEC improves cycleability, what concatenation of reactants may lead to the best SEI, and what other molecules can be used in place of FEC that might be better^17^. In addition, there are many publications showing that FEC benefits Li- and Na-ion battery cathodes and other anodes, not just silicon, but it is similarly unclear the origin of this benefit^18–24^.

It is generally agreed that FEC containing electrolyte produces a more polymeric SEI compared to traditional battery solvents. Furthermore, electron microscopy studies generally report the SEI with FEC to be thinner and more uniform than the SEI without FEC^25–27^. However the exact details, derived from traditional SEI studies such as with XPS, are contradictory and confusing to sort out given the variety of electrode configurations (only Si versus composite) and states of charge investigated. One of the main variations revolves around reported XPS data regarding the concentration of F-containing species (LiF) and the organic chemistry from the polymers^28^. The origin of the LiF could come from either the LiPF_6_ salt or decomposition of the FEC^7, 29^. Uchida *et al*. showed via XPS that the SEI with FEC was far more LiF like than a similar more organic SEI derived from vinylene carbonate (VC) additives indicating that LiF was favorable for battery performance^30^. Similarly Schroder *et al*. reported for binder free Si thin films more LiF in the SEI produced with FEC than from the base EC/DEC (diethyl carbonate) electrolyte^31^. In contrast other studies indicate that less LiF is incorporated within the SEI when using FEC. For example Chen *et al*. reported that the FEC containing SEI layer was only 8.4 at% F versus 24 at% for FEC-Free electrolytes^29^. Similarly Dalavi *et al*. reported the FEC SEI contained 12 at% F vs. 32 at% F without FEC^26^. Nguyen *et al*. reported using 5% FEC in EC/DEC resulted in an SEI that was significantly more organic while 25% FEC resulted in nearly 50/50 organic to LiF^32^. The best cycle life was obtained with 10–15% FEC^32^ indicating that there may be a “sweet spot” in FEC content. Young *et al*. studied the SEI over binder-free electrodes using synchrotron based XPS measurements of delithiated electrodes after 1 or 5 cycles in LiPF_6_ EC, EC/FEC, and FEC electrolytes^33^. They reported EC based electrolyte resulted in an SEI that was LiF poor on the surface but more LiF like closer to the electrode surface^33^. In contrast the LiF was most concentrated on the surface for the EC/FEC electrolyte while the core contained less LiF^33^. Furthermore the EC/FEC electrolyte contained more polymeric-FEC species and was thinner than the pure EC SEI (estimated to be ~10 nm thick) based on Si signals emanating from under the SEI layer and the EC/FEC SEI contained about half the total F content as the EC samples^33^. Etacheri *et al*. reported that the SEI over Si nanowires with FEC resulted in polycarbonates as the major SEI component whereas without FEC the SEI contained more Li_2_CO_3_^27^. In addition the SEI made from FEC containing electrolyte was thinner (~170 nm based on TEM results) and more homogenous than the SEI built without FEC (~220 nm)^27^.

As noted above the SEI with FEC is generally described as more polymeric in nature but the SEI with standard electrolytes is also partially polymeric; the exact compositions are subject to interpretation but there appear to be subtle changes in the composition/speciation with and without FEC. Xu *et al*. investigated the SEI over a Si/CMC/Carbon black composite electrode at various states of charge and after 85 cycles. They showed that the SEI built using FEC contained –CHF-OCO_2_- type polymeric species and different O-C-O and –OCO_2_- compounds than were observed for electrolytes without FEC due to a different coordination structure^34^. Bordes *et al*. reported that over a Si/PAA(poly acrylic acid)/graphene indicate that FEC gives a thinner SEI layer (30–50 nm vs 100–150 nm) with less CH_2_OCHO_2_Li, Li_2_CO_3_, LiF that is more uniform and stable^25^. Dalavi *et al*. in contrast showed the SEI had less C-O species and was dominated by C-C and C-H compounds^26^.

Our lack of understanding of SEI chemistry prevents the development of effective SEI formation processes and materials for Si anodes. As the previously reported results discussed above demonstrate there are variations in the details as to why the FEC works. If one could understand how additives work and correlate to battery performance that would lead to advances in electrolyte design or formation processes. Furthermore, there are relatively few studies of the electrodes at various states of charge^34^, instead of after cycling, missing key insights into the formation of the SEI chemistry. And there is relatively little work to correlate SEI chemistry as a function of oxide termination of the silicon^35, 36^. In this work, we sought to explore some of these issues and provide insight into the role of FEC in SEI formation by characterizing the SEI growth *in situ* using neutron reflectometry (NR) and *ex situ* using infrared and X-ray photoelectron (XPS) spectroscopies. In NR, the specular reflection of neutrons from an interface is measured as a function of the wave vector transfer, *Q* = *4π*sin(*θ*)*/λ*, perpendicular to the sample surface. The angle of incidence *θ* is between the incoming neutron beam and the sample surface, and *λ* is the wavelength of the neutron. Analyzing the neutron reflectivity gives us information about the thickness and composition of the film layers. This ability to follow chemistry and composition has been used to follow the swelling of silicon anodes during alloying/dealloying with Li^37–39^. However, the real power of NR comes from the sensitivity of neutrons to low *z* components (i.e. H, Li, C, O, F), which makes it a good complementary method to X-rays.

We have been exploiting this sensitivity in the last few years to probe SEI chemistry *in situ*. The collected data is analyzed using models built from electrochemical measurements and *ex situ* characterization data and known structures of a battery electrode (i.e. there is always an SEI layer on cycled Si), resulting in unique insights to the SEI thickness and chemistry as a function of state-of-charge and cycle. One key feature of these studies is the use of deuterated solvents to provide contrast at the electrolyte/electrode interface. Protiated solvents, having a lower scattering length density, can produce an SEI with a scattering length density very close to the Li:Si alloy or the electrolyte itself, depending on the state of charge^26^. The low contrast between those layers makes them very difficult to distinguish, which may explain some of the results in the literature^39, 40^.

In our previous studies, we demonstrated that exposing the silicon electrodes to 1.2 M LiPF_6_ EC:DMC electrolyte resulted in a chemical reaction and the resulting dissolution of Si surface (35 Å) resulting in electrode degradation and loss of capacity^41^. *In situ* cycling studies with the same electrolyte revealed the formation of a dynamic 180–300 Å SEI layer that shrinks upon lithiation and expands with delithiation^42^. The chemistry of this layer changes from more inorganic (LiF-like) at fully lithiated states to more organic-like at low lithium content^42^. Subsequent work using deuterated dimethyl perfluoroglutarate solvents showed a similar dynamic SEI structure but revealed that washing the electrode using DMC (standard surface analysis protocol) removed the top 200 Å of the SEI layer consisting of organics^43^. Demonstrating the removal of this SEI component reveals that the SEI chemistry is quite complex and *ex situ* characterization may not capture the total SEI chemistry effectively. In all of our studies at open circuit voltage (i.e. just with electrolyte in contact with the electrode) we always observe the formation of 30–60 Å condensed layer on the electrode surface^41–44^. This condensed layer is due to the segregation of Li ions or Li-rich species and we believe this to be the very initial stages of the SEI formation reaction. This layer is larger than what would be expected for a simple double layer and is not captured by current theoretical models^45, 46^. Beyond this Si work there have been other studies on TiO_2_, copper current collectors, carbon containing anodes^47^ and LiFePO_4_^48^ and LiMn_1.5_Ni_0.5_O_4_ cathodes^44^. These previous studies show SEI layers with thicknesses between 30 and 280 Å depending on the cell chemistry. Finally, recent X-ray reflectivity studies on crystalline silicon indicate a 10 nm inorganic layer at the liquid-solid interface consistent with the ranges reported previously but still unable to probe the organic structure^49^.

In this study we explore the dynamics and chemistry of silicon anodes with FEC containing electrolytes. This work reveals significant changes to the SEI chemistry, compared to non-FEC electrolytes as a function of state-of-charge, and provides evidence for how the FEC improves the SEI chemistry for an amorphous silicon electrode with Si-OH termination.

## Results

**Figure 1:**
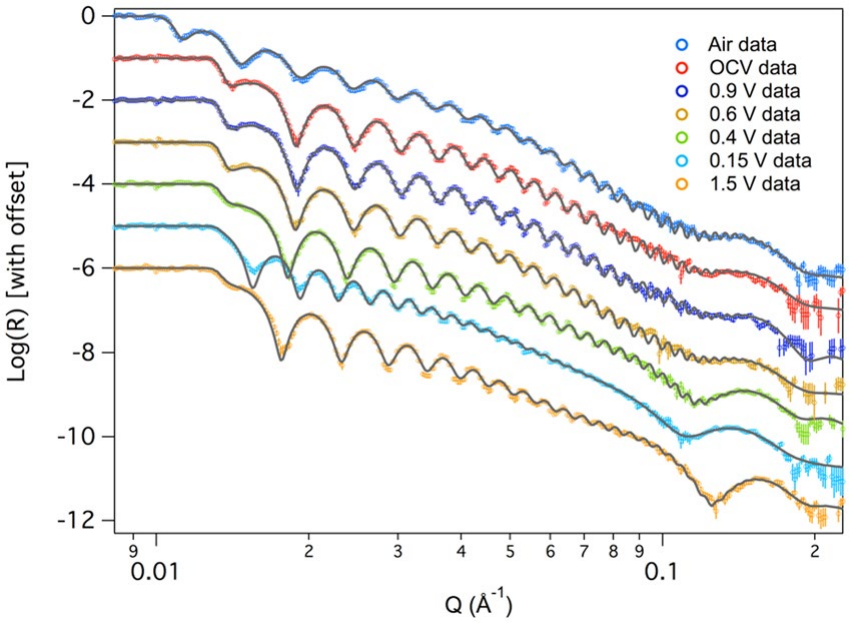
NR data (open data points) and fits (solid line) derived from the SLD profiles for representative data sets collected in this study.

**Figure 2:**
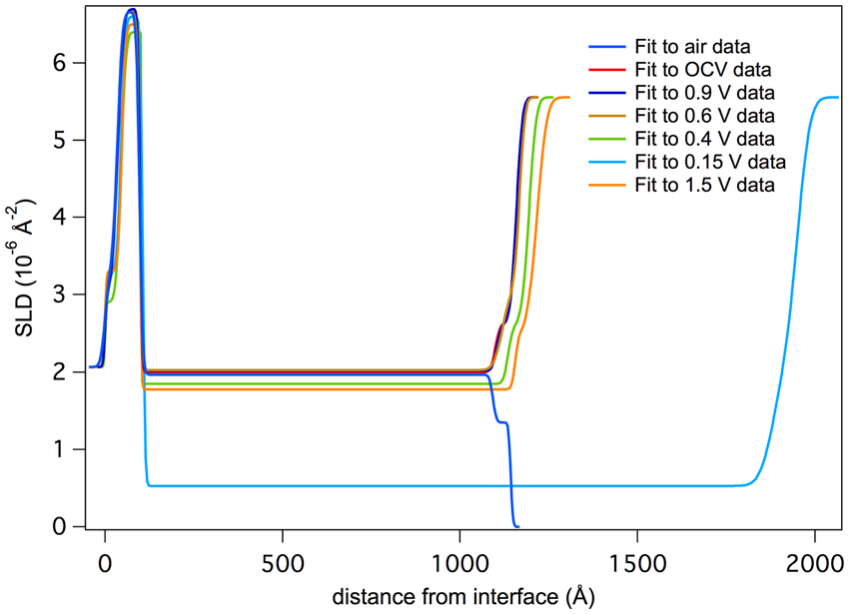
Plot of the refined SLD profile of the film as a function of distance from the thick Si substrate.

**Figure 3:**
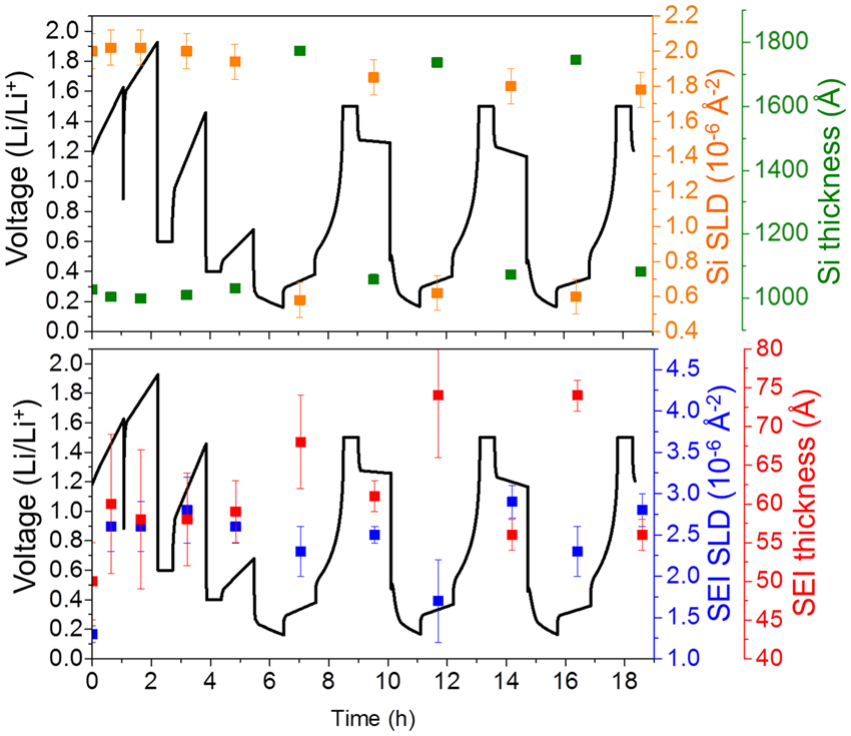
Plots of SLD values and layer thicknesses as a function of state of charge for silicon (top) and the SEI (bottom).

Figure 1 shows representative NR data, along with the fits to the data. Table 1 summarizes the refined values from all the fits while Fig. 2 shows the refined SLD profile of the film at different states of charge. From the data in Fig. 1, it is clear that there are significant changes in the reflectivity profile as a function of cycling, as evident by the changes in fringe spacing and intensity, corresponding to changes in layer thicknesses and compositions. Figure 3 summarizes the electrochemical data with the resulting Si and SEI thicknesses and SLD values.

**Table 1:** Refined thicknesses and SLD values for the sample measured in this study. Si SLD values without errors were constrained using the fitted thickness while those with errors were left to vary in the fit. The values in the SEI columns for the air fit are actually the oxide layer on top of the a-Si.

Li-Si ratio	Voltage	Substrate SiO_x_ Thickness (Å)	SiO_2_ SLD (10^−6^ Å^−2^)	Cu Thickness (Å)	Cu SLD (10^−6^ Å^−2^)	Si Thickness (Å)	Si SLD (10^−6^ Å^−2^)	SEI Thickness (Å)	SEI SLD (10^−6 ^ Å^−2^)	SEI Roughness (Å)	Chi^2^
0.00	air	35 ± 1	3.3 ± 0.2	62 ± 1	6.7 ± 0.1	995 ± 6	2.0 ± 0.1	50 ± 5	1.3 ± 0.1	5.0 ± 0.3	1.7
0.00	OCV	35 ± 1	3.3 ± 0.2	62 ± 1	6.7 ± 0.1	1002 ± 11	2.0 ± 0.1	60 ± 9	2.6 ± 0.3	14 ± 2	2.0
0.00	0.90	35 ± 1	3.3 ± 0.2	62 ± 1	6.7 ± 0.1	1002 ± 11	2.0 ± 0.1	58 ± 9	2.6 ± 0.3	13 ± 1	1.7
0.01	0.60	35 ± 2	2.9 ± 0.4	63 ± 1	6.7 ± 0.1	1009 ± 8	1.93	58 ± 6	2.8 ± 0.4	15 ± 2	1.6
0.03	0.40	45 ± 1	2.9 ± 0.1	59 ± 4	6.4 ± 0.1	1033 ± 5	1.85	59 ± 4	2.6 ± 0.2	16 ± 3	1.4
0.94	0.15	45 ± 1	3.5 ± 0.1	61 ± 1	6.6 ± 0.1	1778 ± 8	0.53	68 ± 6	2.3 ± 0.3	27 ± 3	1.7
0.03	1.50	46 ± 1	3.3 ± 0.1	51 ± 1	6.5 ± 0.1	1056 ± 4	1.78	61 ± 2	2.5 ± 0.1	21 ± 2	2.0
0.97	0.15	47 ± 1	3.4 ± 0.1	56 ± 1	6.4 ± 0.1	1728 ± 12	0.59	74 ± 8	1.7 ± 0.5	30 ± 4	2.5
0.10	1.50	53 ± 1	3.7 ± 0.1	46 ± 1	6.3 ± 0.1	1079 ± 4	1.72	56 ± 2	2.9 ± 0.2	20 ± 2	2.0
1.04	0.15	55 ± 1	3.8 ± 0.1	48 ± 1	6.3 ± 0.1	1753 ± 5	0.56	74 ± 2	2.3 ± 0.3	34 ± 3	2.7
0.15	1.50	64 ± 1	3.8 ± 0.1	35 ± 1	6.7 ± 0.1	1095 ± 5	1.67	56 ± 2	2.8 ± 0.2	23 ± 3	1.9

### As prepared Si electrode

**Figure 4:**
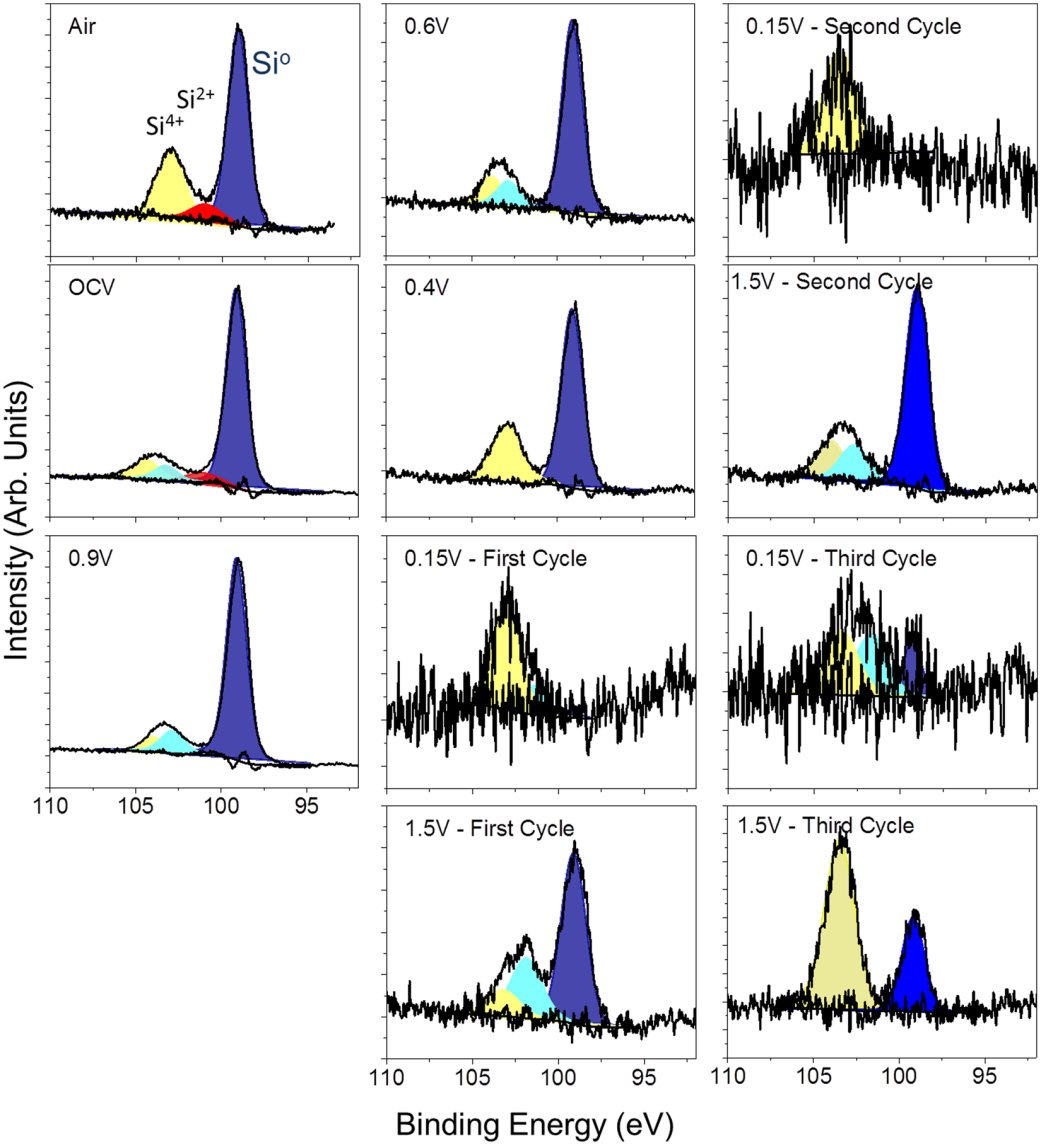
Si2p XPS data collected for the witness samples.

**Figure 5:**
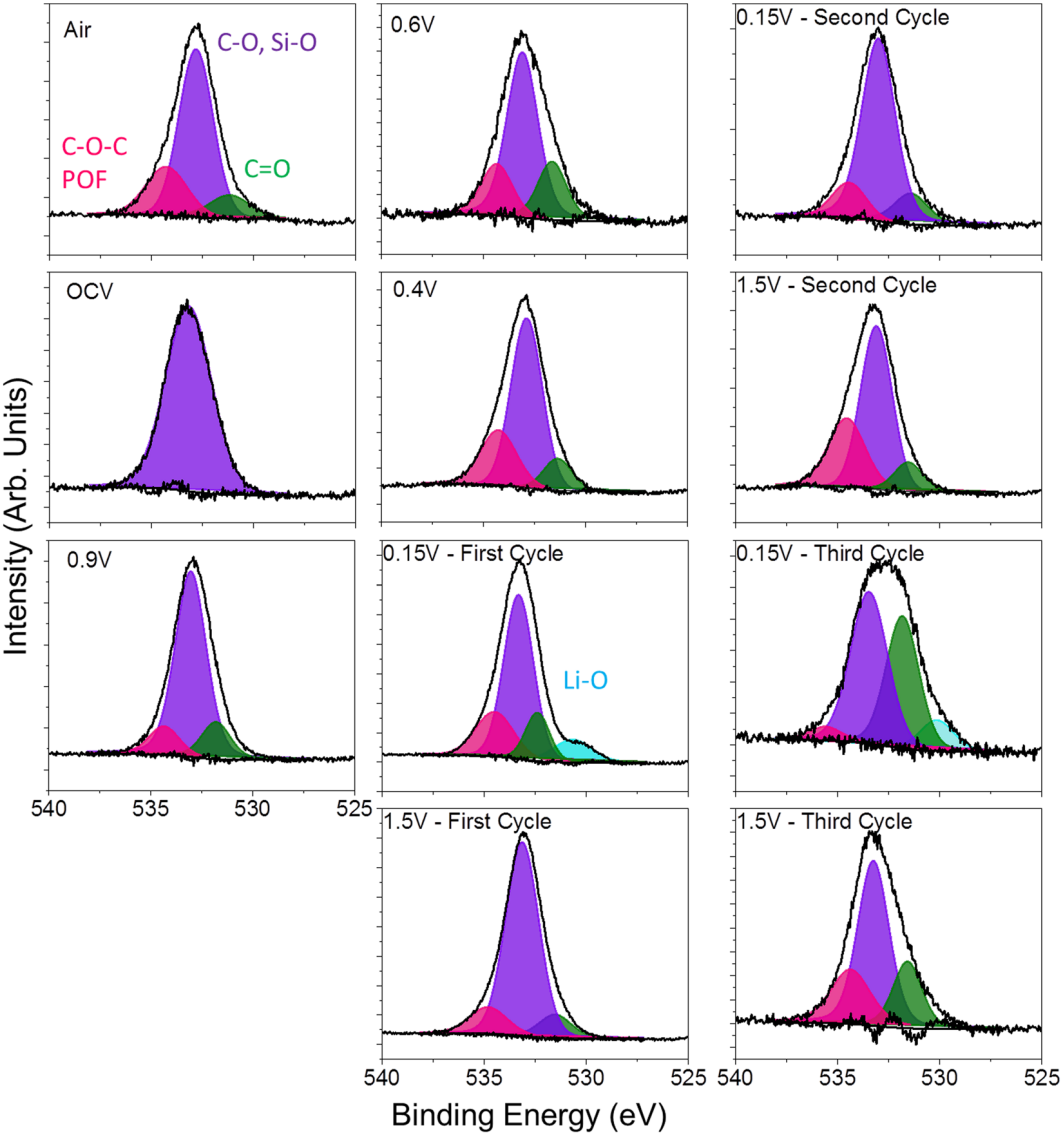
O1s XPS data collected for the witness samples.

**Figure 6:**
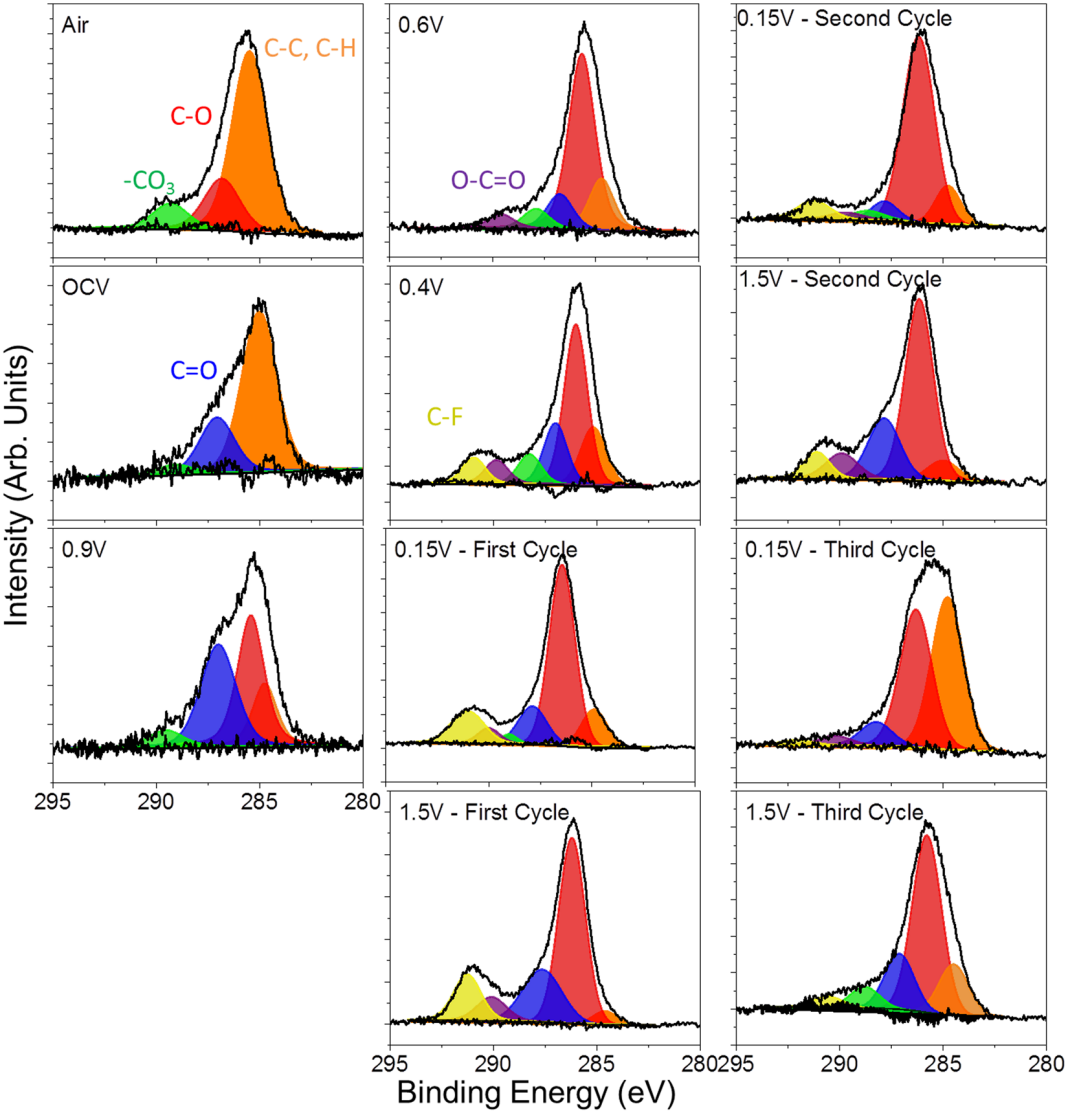
C1s XPS data collected for the witness samples.

Before electrochemical cycling, the *in situ* electrode was analyzed in air by both NR and XPS to develop an understanding of the initial electrode structure. The refinements indicated that the copper electrode was 62 ± 1 Å thick with a SLD of 6.7 ± 0.1, consistent with metallic copper (Note units are 10^−6^ Å^−2^ but are left off for clarity). The refined SLD of the as prepared a-Si layer was 2.0 ± 0.1 which is in good agreement with the theoretically predicted SLD for Si (2.07). The Si working electrode was terminated with a 50 ± 5 Å thick passivation layer consistent with an oxide surface layer as evident in the Si2p XPS data collected for the as-prepared *ex situ* electrode (Fig. 4). In addition, the O1s (Fig. 5) and C1s data (Fig. 6) indicate the presence of C-H or C-C species due to adventitious carbon (284.8 eV) and C-O and –CO_3_ species (286.8 and 289.2 eV respectively and correlated with the O1s data, Table S1) from reaction with the Si-O layer. This passivation layer had a SLD of 1.3 ± 0.1, which is significantly lower than the SLD measured for the native SiO_x_ (3.3 ± 0.2) on the substrate. This reduced SLD is consistent with the presence of surface protons associated with a hydrogen-containing layer that would counteract the high SLD values of O and C. Such a –OH or C-H termination is consistent with the surface chemistry of SiO_x_^50^. From the XPS data we estimate the oxide coating to be around 3 nm in total thickness based on the Si^4+^/Si^0^ intensities.

### Open circuit voltage

After cell assembly, the electrode was in contact with the electrolyte for 90 minutes during the setup and alignment procedure. During this time there is clear evidence for the modification of the silicon surface from the reaction with the electrolyte. Indeed, the SLD of the interface layer increased from 1.3 ± 0.1 to 2.6 ± 0.3 along with a small increase in the layer thickness (50 ± 5 to 60 ± 9 Å). Given that the SLD of almost all of the components of the electrolyte (D, C, O, P, or F) have high bound coherent scattering lengths (6.6, 6.6, 5.8, 5.1, 5.6 fm, respectively) this layer has to have a high concentration of Li or H (from the FEC) that have negative scattering lengths that would account for the relatively low SLD of the layer.

**Figure 7:**
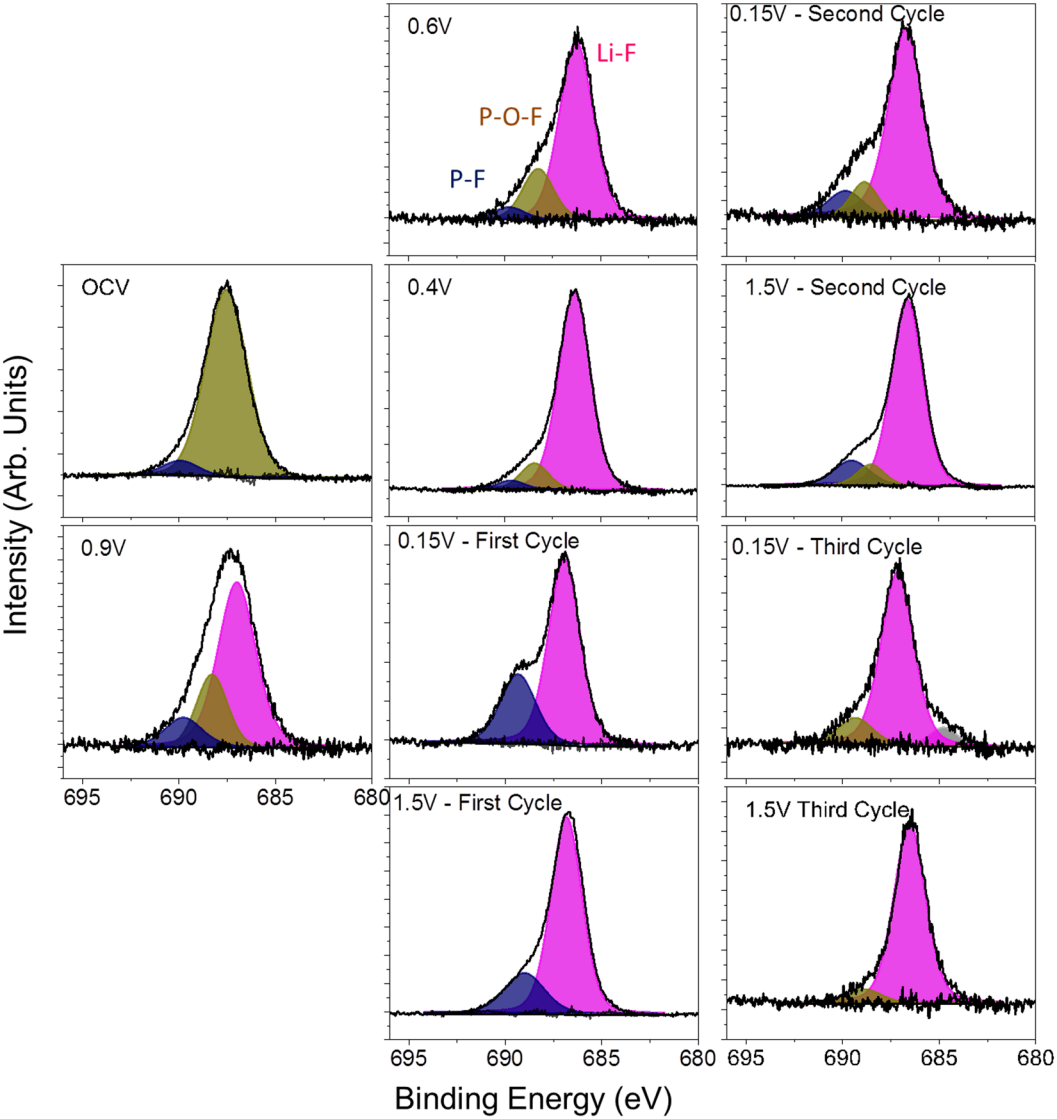
F1s XPS data collected for the witness samples.

**Figure 8:**
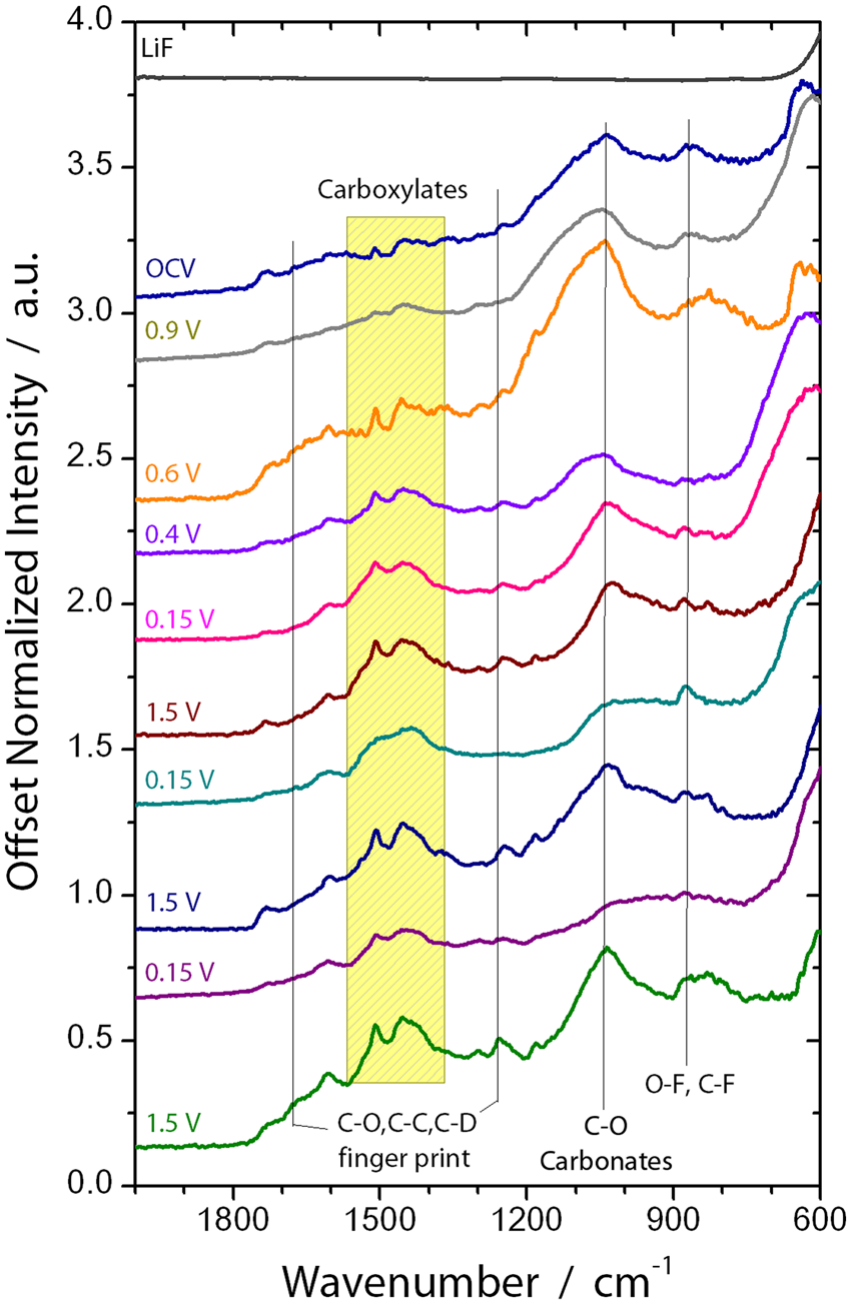
ATR-IR spectra of exact witness samples investigated via XPS.

This change in the surface chemistry is consistent with the measured XPS data where there is a decrease in the concentration of the Si-O bonds at higher binding energies (~104 eV, Table S1) and C on the surface. In addition there is an increase in F (Fig. 7), P and Li (Figures S2 and S3, respectively), which has to originate from the LiPF_6_ or FEC (in the case of F) given the lack of binder in these cells. Analysis of the F1s spectra is consistent with the presence of Li-PF_6_ (690 eV, 6% F species) and Li-P-O-F (687.6 eV, 94% of F species) due to this salt decomposition. The ATR-IR spectrum shows features below 650 cm^−1^ that is reminiscent of Li salts (Fig. 8). Similar P-F and Li-PF_x_ type species are observed in the P2p and Li1s XPS data (Table S1, Figures S2 and S3) as well as the infrared spectrum (broad peaks between 900–800 cm^−1^). In addition there is a change in the C1s spectra (Fig. 6), from the sample exposed to air, where there is the growth of C=O type species (287.1 eV) from the decomposition of the solvent or FEC along with the loss of C-O species. C=O species (presumably in carbonate form) are clearly seen in the ATR-IR spectrum as peaks between 1750 and 1600 cm^−1^. Together this data indicates that the Si-O is being chemically reacted away, likely by the HF originating from the well documented LiPF_6_ reaction with water^51^. But this reaction layer is not simply dissolving, it is being replaced with a new reaction layer formed by the decomposition of salt and solvent species. Such a reaction layer has been observed before for Si electrodes in aprotic solvents and likely represents the initial stages of SEI formation^41–44^. Finally, given the clear evidence for Si^0^ in Fig. 4 this would confirm that the reaction layer is thin consistent with the NR data.

### Before Silicon Lithiation (0.9 V)

The cell was further polarized to 0.9 V (vs. Li/Li^+^), which is a potential above the decomposition of the EC/DMC but expected to reduce FEC. From the NR data there was no change in the Si layer thickness or SLD, consistent with no lithiation. Furthermore, the thickness and SLD of the SEI layer remained unchanged from that of the thickness and SLD of the SEI at OCV. However, there are subtle but important changes in the C1s and F1s XPS data at this potential in the witness samples and faint changes in the IR. In the F1s data there was a clear and distinct formation of a new species with a binding energy of 686.9 eV attributed to the formation of LiF. This is supported by the Li1s data, which shows a peak at 56.6 eV, again consistent with LiF. The C1s data shows the reemergence of a C-O species at 285.3 eV originating from solvent decomposition along with the same previously observed C-C, C-H, -CO_3_ species described previously. This is consistent with the growth of C-O species reported previously and due to decomposition of the solvent^27, 33^. Smaller changes are observed for the P2p data which shows the growth of P-O type species and the loss of P-F components. These changes in the XPS data were further confirmed with ATR FTIR measurements (Fig. 8) that also show the formation of LiF and like salts (evident by the large peak forming just under 600 cm^−1^ and the broad shoulder around 1100 cm^−1^ due to O-C-O and C-O bonding). Given the lack of change in the SEI thickness and SLD, this data indicates an electrochemically driven decomposition/evolution of the 60 Å layer formed at OCV where salt and solvent are decomposed in place forming the electrochemical SEI layer. The lack of difference in the SLD of the SEI layer is due to the similar SLD values of the various SEI components.

### Initial Stages of Lithiation (0.6 and 0.4 V)

With further lithiation, we start to see evidence of changes in the Si electrode chemistry at potentials less than 0.6 V vs. Li/Li^+^. Indeed, at 0.6 V there is a small 8 Å increase in Si thickness (versus as-prepared Si) and a corresponding decrease in the Si layer SLD from 2.0 ± 0.1 to a calculated SLD of 1.93 consistent with Li entering the Si electrode causing the layer to swell and a decrease in SLD due to the negative SLD of Li (−0.87). At 0.4 V the thickness increased by 24 Å and the SLD decreased further to 1.85, again consistent with the extent of lithiation and the expected swelling of Li:Si alloys reported by Chevrier, Figure S3 ^52, 53^.

At these stages of lithiation there is no apparent change in the SEI SLD or thickness based on the NR data. However, there are clear changes in the XPS data again due to the formation of more C-O species (~285.5 eV) and O=C-O moieties (~288 eV), from the breaking of C=O bonds in the solvent or polymerization of the cyclic carbonates, and additional LiF at the expense of residual Li-P-F and Li-P-O-F species. This is confirmed with the decrease in the broad IR bands at between 900 and 850 cm^−1^. In addition, we see evidence for a higher binding energy C1s species (290 eV) attributed to C-F species due to the reduction of FEC. The presence of these species is confirmed by IR with the increase definition of the peak between 1300–1100 cm^−1^. Interestingly, there is a second lower energy Li1s peak (56.2 eV) evident in the XPS data measured for the 0.4 V sample in addition to the main LiF peak. This new feature corresponds to Li-Si bonding in the anode that is detectable due to its concentration and the thin SEI (59 ± 4 Å), which does not attenuate the excited photoelectrons. This data supports the validity of the SEI thickness derived from the analysis of the NR data. Finally, this data differs from previous XPS reports^14^ of FEC based electrolytes over Si (100) electrodes which were dominated by C-C/C-H species, no C-O bonds, indicating a different reaction mechanism over crystalline Si versus the amorphous Si here.

### Cycling of Li-Si anodes (0.15–1.5 V)

NR data collected for the silicon anode with extensive Li cycling revealed significant and dynamic changes in the SEI thicknesses and composition. The electrodes were repeatedly cycled from a Li-Si stoichiometry close to LiSi to a Li_0.1_Si (Table 1). Upon lithiation the electrode swelled to about 1778 ± 8 Å (from 995 ± 6 Å), causing a significant decrease in the Si SLD; during delithiation the Si returned to a thickness of 1056 ± 4 Å due to slight irreversibility of the cell. The fitted SLD values and thicknesses determined for the silicon layer were in good agreement with the predicted values based on the electrochemical measurements, Figure S4. Furthermore, the XPS data collected on these electrodes revealed the Si2p spectra still contained information demonstrating the presence of reduced Si from the electrode. Together this data confirms that the SEI is less than 10 nm thick and again supports the validity of our NR model.

The more interesting data was obtained for the SEI layer. As demonstrated in Fig. 3, the SEI layer increased in thickness by about 12–13 Å with lithiation, and its SLD decreased to about 2.3 ± 0.3 (from 2.6 ± 0.3). Upon delithiation the SEI shrunk to its prelithiation thickness and the SLD increased to 2.5 ± 0.1. This swelling and contracting of the SEI layer thickness is consistent with the “breathing” reported for silicon anodes measured by previous NR studies as well as XPS, TOF-SIMS, and atomic force microscopy studies^31, 34, 42, 43, 52, 54^. We should note that in the case of the FEC, the thickness of the SEI “breathes” in the opposite direction of the non-FEC case. The expansion upon lithiation with FEC would indicate the electrolyte continues to decompose as the layer expands. Given the apparent increase in organic signal and the higher SLD this would indicate solvent decomposition.

**Figure 9:**
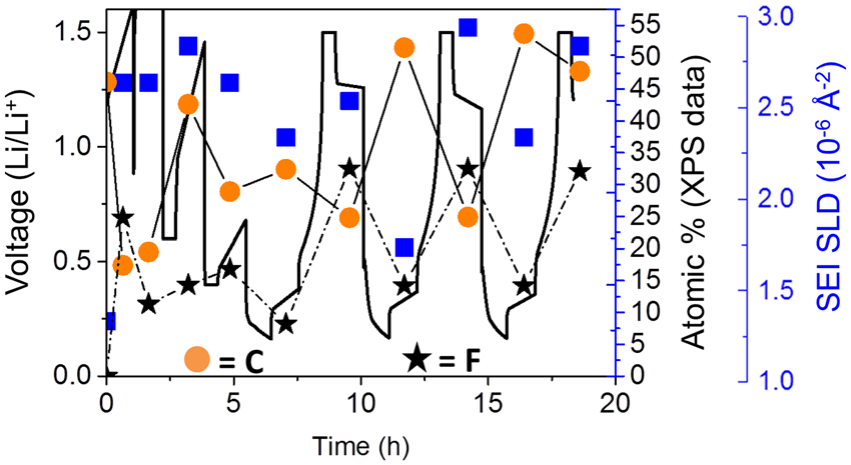
Plot of C and F atomic concentrations as a function of state of charge and SEI SLD.

XPS analysis of the Si electrodes at various states of charge reveal significant changes in the SEI chemistry with cycling that follow the changes in SEI SLD that occur with the electrode “breathing”. Figure 9 shows a graphical representation of the atomic concentrations of C and F as a function of state of charge and SLD measured for the SEI. The C concentration data is a surrogate for the organic portion of the SEI while the F signal (originating primarily from LiF) represents the inorganic portion of the SEI. From this data it is clear that at states of high lithiation (0.15 V) the organic fraction of the SEI increases while the inorganic fraction decreases. Upon delithiation the concentration of the species reverses and the inorganic fraction of the SEI dominate the SEI layer while the organic content is reduced. This data indicates that the growth in the SEI thickness with lithiation originates from the further decomposition of organic solvent molecules resulting in a more organic layer as the Si swells. Upon delithiation this deposited organic layer is decomposed off or dissolves into the electrolyte. The addition of Li and H (from the FEC) drives the SLD of the SEI down during lithiation, while the removal of these species leads to the increase in SLD during delithiation.

The “breathing” is also seen in the IR spectra. Interestingly, the spectra at 0.15 V show less features than those at 1.5 V. The P-O-F region (900-800 cm^−1^) is subdued at 0.15 V, though it shows more small peaks in that C-D/C-H finger print region. At low state-of-charge, the poly-carbonates and lithium salts are more clearly defined. The broad peak between 1100 and 1000 cm^−1^ is due to a wide range of C-O and carbonate stretches. It is particularly sensitive to the state of charge and agrees with the XPS result where the C-O is far more prevalent than C-H at 1.5 V.

Fitting the individual spectra collected for each sample reveal additional changes to the SEI chemistry of both the organic and inorganic components. The C1s data, originating from the organic component (Fig. 6), shows the resulting SEI is still dominated by C-O bonding (~286 eV), from the polymerization of the cyclic carbonates or polymerization of the C=O bonds from the solvent, along with small concentrations of C-F (~291 eV) from the decomposition of the FEC, C=O, O-C=O, -CO_3_, and C-C/C-D from the decomposition of the solvent. The presence of the carbon-oxygen moieties is supported/confirmed by the O1s data (Fig. 5). The spectral contribution from each of these species stays relatively constant with cycling, only the absolute concentration changes. This data confirms the dissolution of the organic species upon delithiation. In addition the O1s data shows for the 0.15 V data the growth of a new species with a binding energy around 530.2 eV attributed to the formation of inorganic Li-O. This Li-O has been reported previously^3^. Interestingly this Li-O disappears during charging indicating the possible formation of a reactive oxygen species which is known to decompose organic electrolytes^55, 56^.

The F1s data, originating from the inorganic components, shows a more dynamic chemistry than the C containing components. For all the samples the F1s spectra is dominated by Li-F type species. However, with lithiation there appears to be an increase in P-F components due to decomposition and trapping of the LiPF_6_ salt (evident in the high binding energy F1s species (~689.5 eV). This is confirmed with the P2p data (Figure S2), which shows more P-F species (~137.6 eV) at 0.15 V. Upon charging the P-F revert to more P-O-F (688.3 eV for F1s and ~135 eV for P2p data). Together this indicates a continuous decomposition of the Li salt in addition to the solvent molecules described above.

Finally, the NR data revealed changes in the Cu-SiO_x_ interface with cycling. The data in Table 1 shows the thickness of the copper and SiO_x_ layer as well as their SLD values. During the electrochemical cycling of Li the potential was sufficient to drive the co-diffusion of Cu and SiO_x_. This is consistent with previous studies on memory devices^57, 58^ and does not influence the electrochemistry of the Si/SEI discussed above, but it may influence data analysis for those seeking to perform similar neutron studies with this level of resolution or similar X-ray studies.

## Discussion

**Figure 10:**
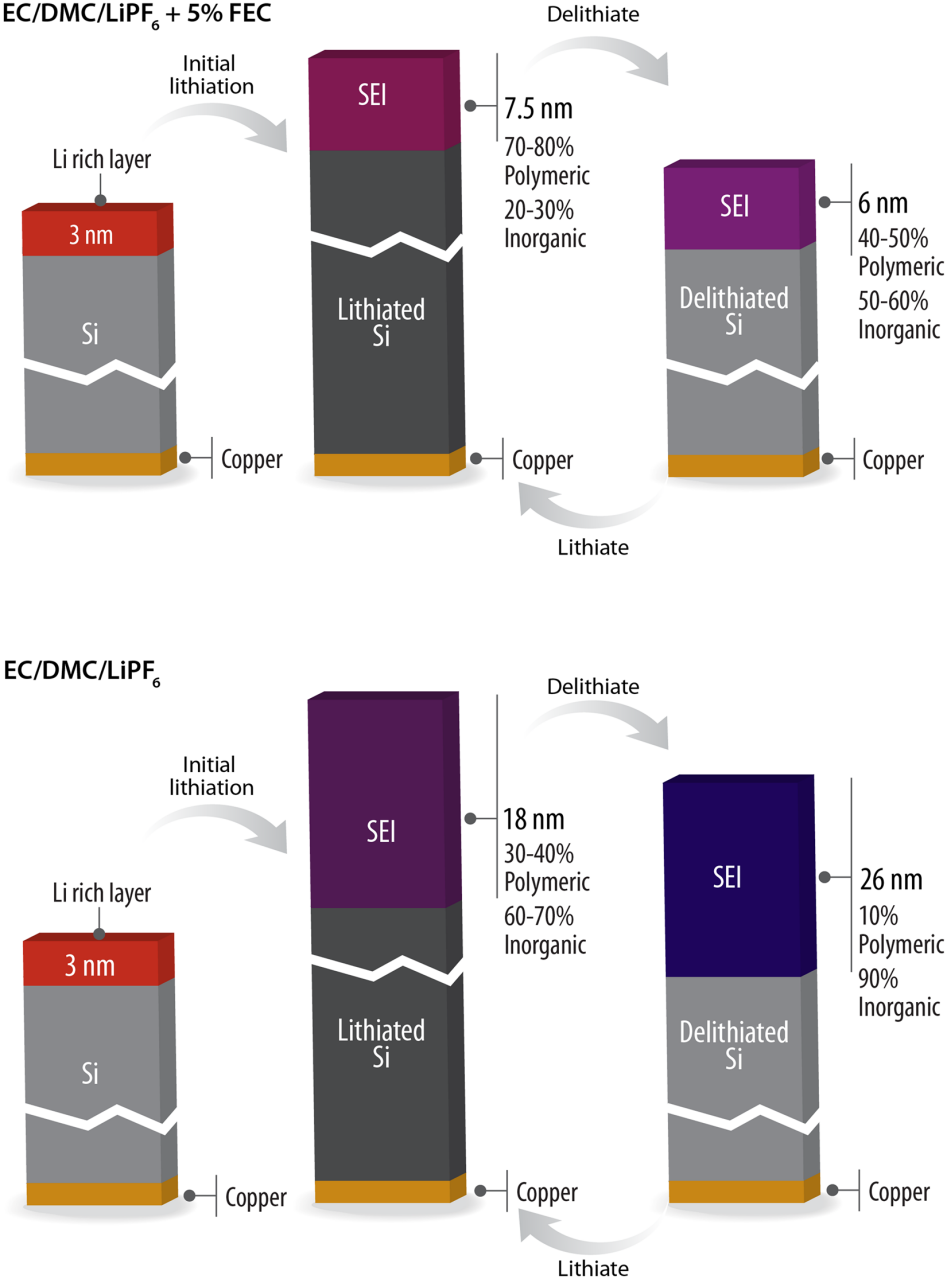
Graphical summary of SEI layer chemistry grown on silicon with and without FEC.

The above data demonstrates the structure and composition of the SEI formed over a silicon anode with lithium cycling in an electrolyte that contains FEC as an additive. At this point we will compare this data with previously reported NR data collected for electrolytes without FEC in the same electrochemical experiment. The SEI thickness and SLD values for these two samples are replotted in Figure S5 to aid in the comparison and summarized graphically in Fig. 10. The OCV for both sets of data show a similar condensed layer with a thickness of about 50 Å, indicating a similar starting point to SEI formation. However with cycling the previous, non-FEC containing electrolyte, data showed the immediate formation of SEI layer with a thickness between 180 and 250 Å. This SEI layer shows a similar “breathing” as is observed for the data in this publication, however the magnitude of the breathing (60 Å) is significantly larger than the 13 Å measured for the FEC containing electrolyte. Interestingly the direction of this “breathing” seems to change between the different electrolytes. With the FEC the layer gets thicker with lithiation while without FEC the layer becomes thinner. In both cases the SEI becomes more polymeric with lithiation and less polymeric with delithiation. The key to this difference appears to be the compositions which vary as a function of state of charge. The FEC SEI is more polymeric (70–80%) versus 30–40% during lithiation. This polymer content grows versus the delithiated state (40–50 vs. 10% - respectively). In the case of FEC this increase in SEI thickness during lithiation is likely due to the continued decomposition of solvent. In contrast without FEC the less polymeric SEI does not prevent further LiPF_6_ decomposition during delithiation growing the inorganic LiF component. Upon relithiation polymer species reform and displace the LiF layer which likely breaks off due to its mechanical stiffness during the electrode expansion or the dissolution of polymer/solvent species in the SEI.

The thickness of the SEI measured with NR is less than what was observed for a composite electrode reported by Xu *et al*. (>15 nm) during lithiation however, the SEI was observed to thin upon delithiation^34^, similar to what we report here. This may indicate additional reactions promoted by binders and carbon additives influencing SEI chemistry. Reported Time-of-Flight Secondary Ion Mass Spectrometry (TOF-SIMS) data indicates that the SEI grown over a silicon thin film with FEC when delithiated was about 6.6 nm thick in good agreement with these results^31^. The TOF-SIMS data for the same electrode lithiated shows an SEI that is 35.1 nm thick^31^. This is consistent with our results, though we note the actual SEI thickness in this report^31^ is probably quite a bit thinner than the 35.1 nm as evident by the Si2p signal from Si^0^ evident in their XPS data for their sample likely due to an artifact of a different sputter rate for the two SEI layers due to changes in the SEI chemistry organic/inorganic composition.

Comparing the SEI compositions of the two NR studies reveal that the non-FEC containing electrolyte SEI (at 0.12 V vs. Li/Li^+^) was comprised of similar functionality (i.e. C-C/C-H, C-O, C=O, O-C=O, -CO_3_, LiF, POF, etc.), but the organic content comprised about 4 at% carbon. There was a correspondingly larger increase in inorganic F-containing species (~80% LiF) in non-FEC electrolyte. This is significantly less organic components than the SEI produced from FEC containing electrolyte. These results are in good agreement with previous XPS studies which show a more organic SEI with FEC^14, 25–27, 31–34^ and NMR studies showing more LiF without FEC^59, 60^.

Together this data provides insights into how FEC aids in the cycling of Si anodes for use in Li-ion batteries. The preferential reduction of FEC at potentials greater than 0.9 V (vs. Li/Li^+^) forms a more organic C=O containing polymeric surface layer. This layer is not the SEI but the SEI precursor which further reacts at 0.6 V to form a layer with more C-O species from the polymerization of cyclic carbonates (likely from FEC)^14, 23–25, 28–31^. The SEI layer formed during charging from the electrolyte without FEC is comprised from more inorganic LiF like components. The polymeric FEC electrolyte effectively keeps the SEI from growing to the ~200 Å measured for cells without FEC.

Despite this clear improvement in SEI chemistry, there are still problems evident with the FEC. In both cases the “breathing” measured for the SEI originates from the consumption of electrolyte. This consumption lowers the cycle life of the electrode by consuming the electrolyte needed to move Li ions. The thinner FEC SEI and the much reduced “breathing” of the FEC SEI results in less electrolyte consumption resulting in the reported increase in cycle life of Si based electrodes. However, even these batteries fail with time as the FEC gets consumed. This data would indicate that a more flexible SEI built from polymeric like C-O functionality that form at much higher potentials (vs Li/Li^+^) may provide a pathway to improve the cycleability of Si anodes.

In summary, following the SEI formation using *in situ* neutron reflectometry revealed detailed insights to the role FEC plays in the formation of a stable SEI over silicon anodes. The FEC selectively binds and reacts to the silicon surface at potentials around 0.9 V forming a polymer rich condensed layer on the silicon surface as evident in the XPS data showing the initial formation of LiF (~16% total surface) and C-O from the solvent. In contrast without FEC the layer becomes more LiF like. Upon further lithiation the chemistry evolves from a clear polymerization of the C=O functionality to more Li-O-C-D polymeric species with a low concentration of LiF (~14 at% versus 80% without FEC). Furthermore the FEC based SEI is only ~70 Å thick versus ~200 Å without FEC. Together this indicates that the FEC based electrolytes form thinner SEI and are built from more flexible polymeric components. This thin polymeric layer likely has the flexibility to bend and readjust that LiF based SEI layers would not. This flexibility ensures a more stable SEI that would more effectively passivate the surface against further reactions. Although the FEC based SEI is still not perfect as it changes in thickness and composition with cycling (0.15–1.5 V). Specifically, the layer becomes more organic at high states of lithiation and more inorganic at low states of lithiation due to dissolution of the polymer. This points to a potential path to make a more stable SEI through the formation of a more cross-linked, less soluble polymer SEI possibly through the design of more elaborate, large molecule, fluorinated carbonates. It would be interesting to explore this chemistry as a function of oxide surface termination and polymer binders which likely also participate in the SEI passivation reaction and change the reaction mechanism(s).

## Methods

The thin film working electrode for NR studies was deposited on a 51 mm diameter silicon substrate (Institute of Electronic Materials Technology, Warszawa, Poland) by magnetron sputtering and electron beam evaporation at the Center for Integrated Nanotechnologies at Los Alamos National Laboratory. This was accomplished by using a purpose-built two-chamber load locked system that allows the substrate to be processed first in the magnetron sputtering system and then passed to the electron beam evaporation system while remaining under high vacuum. The Cu layer was deposited via magnetron sputtering at a process pressure of 3 mTorr with an argon flow of 30 sccm. The target had an applied power of 300 watts with a deposition rate of 0.7 nm/s. The sample was then transferred into the electron beam evaporation chamber and the a-Si layer was deposited at a rate of 0.25 nm/s, also at room temperature. Thin film electrodes for *ex situ* X-ray photoelectron spectroscopy (XPS) and Fourier transform infrared spectroscopy (FTIR) studies were deposited on battery grade copper foil by magnetron sputtering from a commercially available Si (99.99% - Kurt J. Lesker) target in an in-house (Oak Ridge National Laboratory) sputtering system. The Si films were deposited at an applied power of 90 W, at 7.5 mtorr Ar (99.9995%, Air Liquide) for 2 minutes.

Battery electrolyte was homemade using components purchased from Sigma Aldrich. 99.9% deuterated EC and deuterated DMC were mixed in a well-dried vial in a 30:70 wt% ratio in an argon filled glove box. The solvent mixture was dried with zeolites for 2 months to remove water and small strips of Li to react with impurities. The exact water content was not determined. 4 hours before use, the solvent was mixed with LiPF_6_ salt (99.99% Battery Grade) to form a 1.0 M electrolyte and 5 wt. % fluorinated ethylene carbonate (Aldrich 99.9%) to form the final electrolyte. The Li counter electrode was prepared using Li-foil (Alfa Aesar – 0.75” wide), which was scraped to remove surface passivation and pressed onto a machined TiZr substrate in an argon filled glove box (discussed below). The TiZr substrate was previously cleaned in water/isopropanol/hexane baths and dried at 120 °C for 2 days.

The electrochemical cell was constructed using a single crystal Si substrate with a deposited Cu/a-Si film (Si) as the working electrode and the Li-coated TiZr as the counter electrode (half-cell type configuration). A schematic is shown in the supplemental section (S1). TiZr (Ti_0.47_Zr_0.53_) was selected as the Li current collector for several reasons. First, TiZr is a null scatterer of neutrons and would not contribute to the NR spectrum. Second, TiZr is stable in contact with metallic Li and does not react electrochemically with Li. Long wires were bound to the Si and TiZr plates using silver epoxy (Illinois Tool Works), which was allowed to dry overnight. The silver epoxy was placed on the edge of the Si wafer to contact the Cu, which was spilled around the wafer during deposition. The epoxy was never in contact with the electrolyte. The components were assembled in a He filled glove box located at the beamline (H_2_O < 1 ppm; O_2_ < 7 ppm). The electrodes were separated by a Teflon coated Viton O-ring with a 0.8 mm cross sectional diameter. The total cell volume is approximately 2.5–2.8 ml depending on the amount of Li on the TiZr. The electrochemistry was controlled using a Biologic VSP potentiostat. Cells were cycled at a rate of ~C/3 based on the theoretical capacity of the Si working electrodes (Li_4.4_Si) except during the first lithiation step, from OCV to 0.9 V, where rates approaching 0.6 C were used to purify the electrolyte^42^. Electrodes were charged to various potentials and allowed to equilibrate for 10 minutes prior to NR data collection. Voltages are reported with respect to Li/Li^+^. The total current was used to confirm the refined Li-Si ratios discussed below.

NR measurements were carried out on the Liquids Reflectometer (LR) at the Spallation Neutron Source (SNS) at Oak Ridge National Laboratory. The LR is a horizontal geometry instrument using the time-of-flight technique with an effective single bandwidth of 3.5 Å at an accelerator pulse frequency of 60 Hz. For these measurements, neutrons of wavelength 2.5 Å to 17 Å, together with four incidence angles θ = 0.60°, 0.69° 1.19° and 2.34°, provided a wave vector transfer (*Q*_*z*_) range extending from 0.008 Å^−1^ to 0.20 Å^−1^. An incidence beam slit was adjusted for each incident angle in order to maintain a constant footprint on the sample.

### NR data analysis

In this work the films consist of 5 mm thick Si substrate with its native SiO_x_ passivation layer, a copper current collector, the amorphous Si working electrode, the SEI layer and the bulk electrolyte. Reflectivity data were fitted to determine the thickness and scattering length density (SLD) of the various layers comprising the film. The SLD profile represents the composition and density of the layers normal to the film surface and can be represented mathematically by ${SLD}(z)=\sum _{i}{b}_{i}{n}_{i}$ where *b*_*i*_ is the coherent neutron scattering length and *n*_*i*_ the nuclear number density of a given atomic species at depth *z* within the film. The SLD is measured in units of 10^–6^ Å^−2^. For simplicity we will not repeat the units of SLD.

The reflectivity data was obtained from the raw data using the Mantid reduction software^61^. The measured reflectivity was modeled using the Motofit program^62^, following the procedure described in previous studies^42^. An additional constraint was added to the fitting procedure described in the above reference to make use of the established relationship between the SLD of the Li_x_Si layer and its thickness^40, 53^. For this purpose, the measured thickness of the silicon electrode is used to obtain the ratio *x* in Li_x_Si (eq. 1):

1 $$Li:Si\equiv x=\frac{D-{D}_{0}}{{D}_{0}}\frac{{V}_{Si}}{{V}_{Li}}$$

where *D* is the silicon thickness, *D*_*0*_ is the initial silicon thickness before lithiation, *V*_*Si*_ is the volume of the Si atom, and *V*_*Li*_ = 14.7 Å^3^ is the increase in volume for each Li atom added to the Si volume^53^. The SLD of the silicon electrode can then be obtained with eq. 2:

2 $$SLD(L{i}_{x}Si)=\frac{{b}_{Si}+x{b}_{Li}}{{V}_{Si}+x{V}_{Li}}$$

where *b*_*Si*_ and *b*_*Li*_ are the coherent scattering lengths of the Si and Li, respectively.

Before measuring the film in the cell, a reflectivity measurement was done on the as-prepared film in air to determine the initial amorphous silicon thickness (*D*_*0*_ above) and SLD. The value of *V*_*Si*_ in the equation above was then determined using the amorphous silicon SLD measured in air. While modeling the film for our *in situ* measurements, the SLD of the silicon electrode was fixed according to its thickness using the equations above, thus removing the SLD of the silicon as a fit parameter.

The fit results obtained were also validated using the Refl1D fitting software^63^. Like Motofit, Refl1D also uses the Abeles formalism^64^ to model the reflectivity from a layered film. It uses a Markov Chain Monte Carlo approach to minimization and produces a probability distribution for the model parameters^65^. Uncertainties can be extracted from those probability distributions. We compared the output of Motofit and Refl1D for all our fits and found them to be in good agreement within errors.

### *Ex situ* electrochemical experiments

Silicon electrodes grown on copper foil were used for *ex situ* analysis. 13 mm disks were punched out from the coated copper foil and dried under vacuum prior to assembly into coin cells. The coin cells (Pred Materials) were assembled in an argon filled glove box using the same electrolyte and Li used in the NR experiment. The volume of electrolyte was scaled to reflect the change in surface area between the two electrochemical cells. Four pieces of DreamWeaver separator (Gold 40) were used to contain the electrolyte. The cells were cycled using the same current densities and rest times used in the NR measurements. The cells were stopped at the potentials used in the NR studies and disassembled in an argon filled glove box. The working electrode was soaked in 3 mL of dimethyl carbonate for 1 minute and dried under vacuum at room temperature for 25 minutes. The samples were load locked into a PHI 3056 XPS to avoid air exposure. Data were collected using an Al X-ray anode source operated at 15 kV and an applied power of 350 W. High resolution spectroscopy data were collected using a 23.5 eV pass energy; lower resolution survey scans were collected at a pass energy of 93.5 eV. Charge correction was implemented by shifting all the peaks relative to the Si^0^ peak measured for the Si2p excitation (99 eV) though even with this charge correction there was an incommensurate charging that occurred with lithiation which caused the insulating LiF peaks to appear shifted to higher energies. The shifted data (relative to Si^0^) is presented but the presence of the LiF was confirmed with the IR results discussed below^66^.

Infrared spectra were obtained in attenuated total reflection (ATR) mode on a Bruker ALPHA FTIR in an Ar(g) atmosphere glove box with the sample against a germanium window. Randomly selected samples were further investigated by taking multiple spectra around the electrode to probe any differences due to nonuniform current distribution. No evidence of any anisotropy was found. Due to the multilayered nature of the sample, no ATR correction was applied to the spectra. XPS and FTIR data were collected on the *ex situ* films after the cycling of Li. This data confirmed the surface chemistry of the SEI layer and provided a basis to estimate the SEI composition. The charge correction applied to the films was often insufficient for the cycled electrodes due to the incommensurate charging that occurred for the Li-F species resulting in a slight shift to higher binding energies for the Li1s and F1s data^66^. The presence of the LiF was confirmed by the FTIR studies and indicates that these are not different Li and F containing species than LiF.

## Electronic supplementary material

Supplementary information
