# Supplementary material for: Determination of the Solid Electrolyte Interphase Structure Grown on a Silicon Electrode Using a Fluoroethylene Carbonate Additive
Source: Sci Rep. 2017 Jul 24;7:6326. doi: 10.1038/s41598-017-06555-8 (PMC5524684; doi:10.1038/s41598-017-06555-8)
Supplement: Supplementary file 1 — Supplementary information [file 41598_2017_6555_MOESM1_ESM.doc]

**Supplemental** Determination of the Solid Electrolyte Interphase Structure Grown on a Silicon Electrode Using a Fluoroethylene Carbonate Additive

Gabriel M. Veith^1,#,*^, Mathieu Doucet^2,#,*^, Robert L. Sacci^1^, Bogdan Vacaliuc^3^, J. Kevin Baldwin^4^, James F. Browning^5,#,*^

^1^ Materials Science and Technology Division, Oak Ridge National Laboratory, Oak Ridge TN 37831

^2^ Neutron Data Analysis and Visualization Division, Oak Ridge National Laboratory, Oak Ridge TN 37831

^3^ Research Accelerator Division, Oak Ridge National Laboratory, Oak Ridge, TN 37831

^4^ Materials Science and Technology Division, Los Alamos National Laboratory, Los Alamos, NM 87544

^5^ Chemical and Engineering Materials Division, Oak Ridge National Laboratory, Oak Ridge TN 37831

^*^ - Corresponding Authors – [veithgm@ornl.gov](mailto:veithgm@ornl.gov), [doucetm@ornl.gov](mailto:doucetm@ornl.gov), [browningjf@ornl.gov](mailto:browningjf@ornl.gov)

^#^ - These authors contributed equally to this work


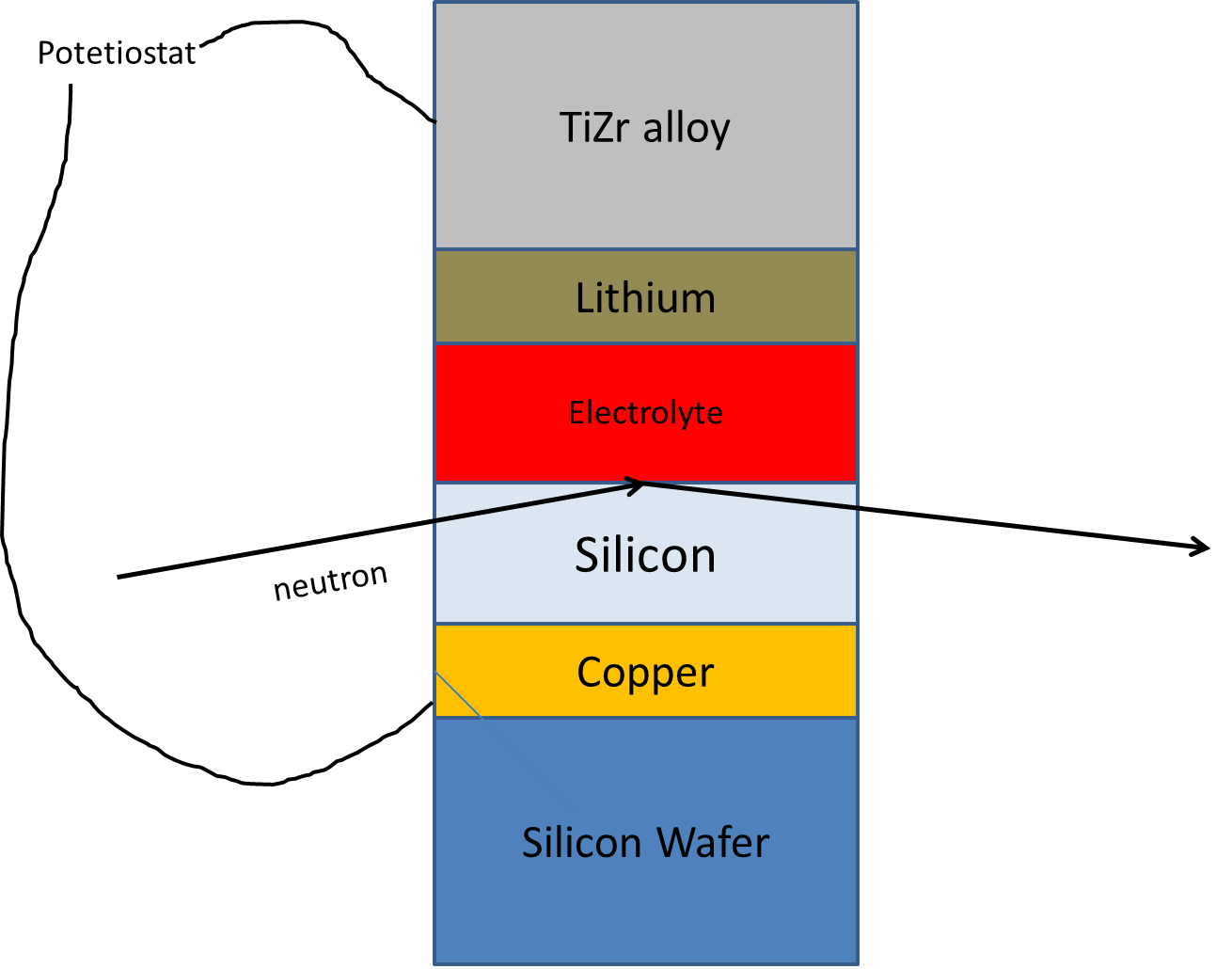


Figure S1. Schematic of NR cell

Figure S2. P2p XPS data collected for these materials


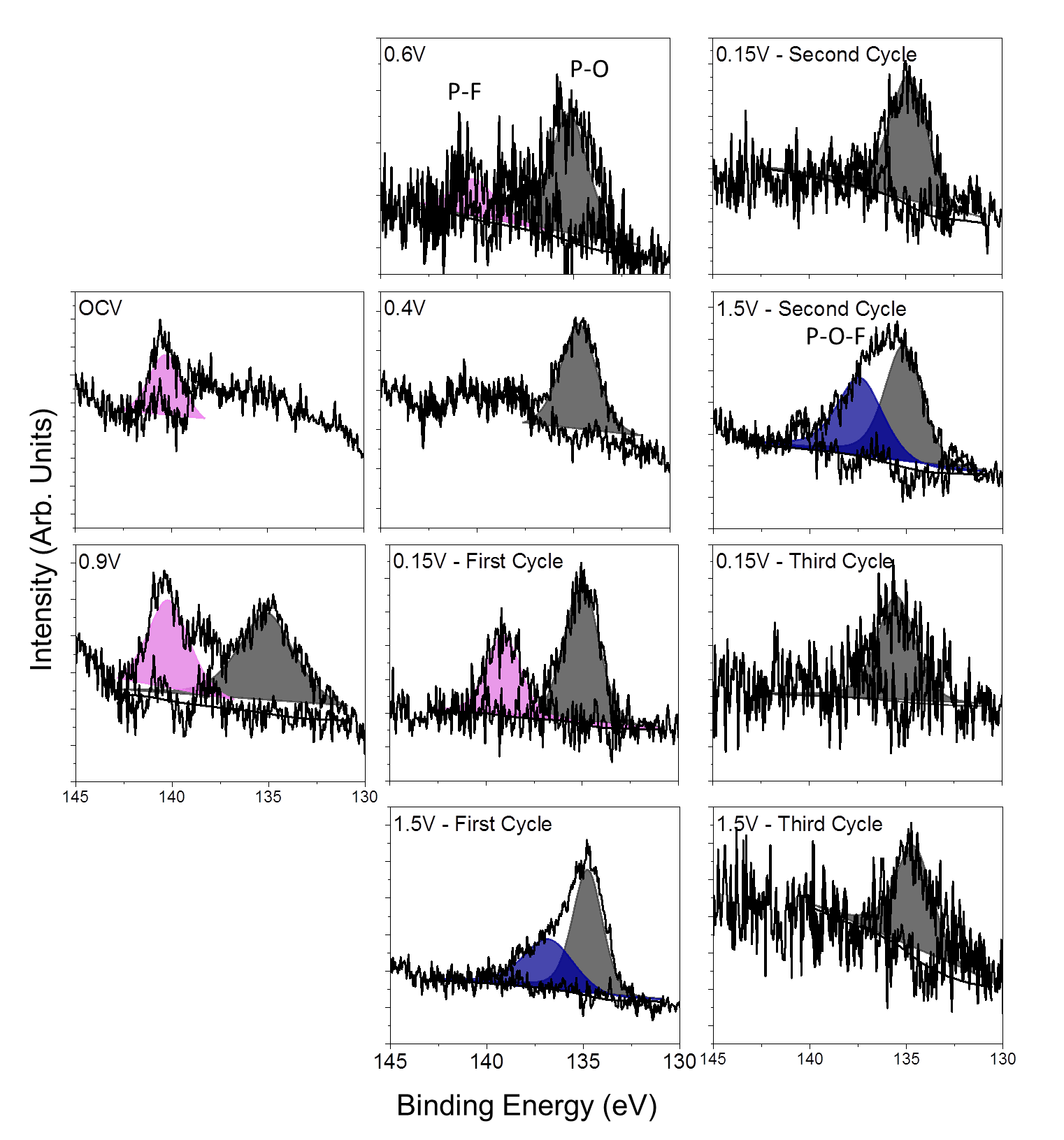


Figure S3. Li1s XPS data collected for these materials


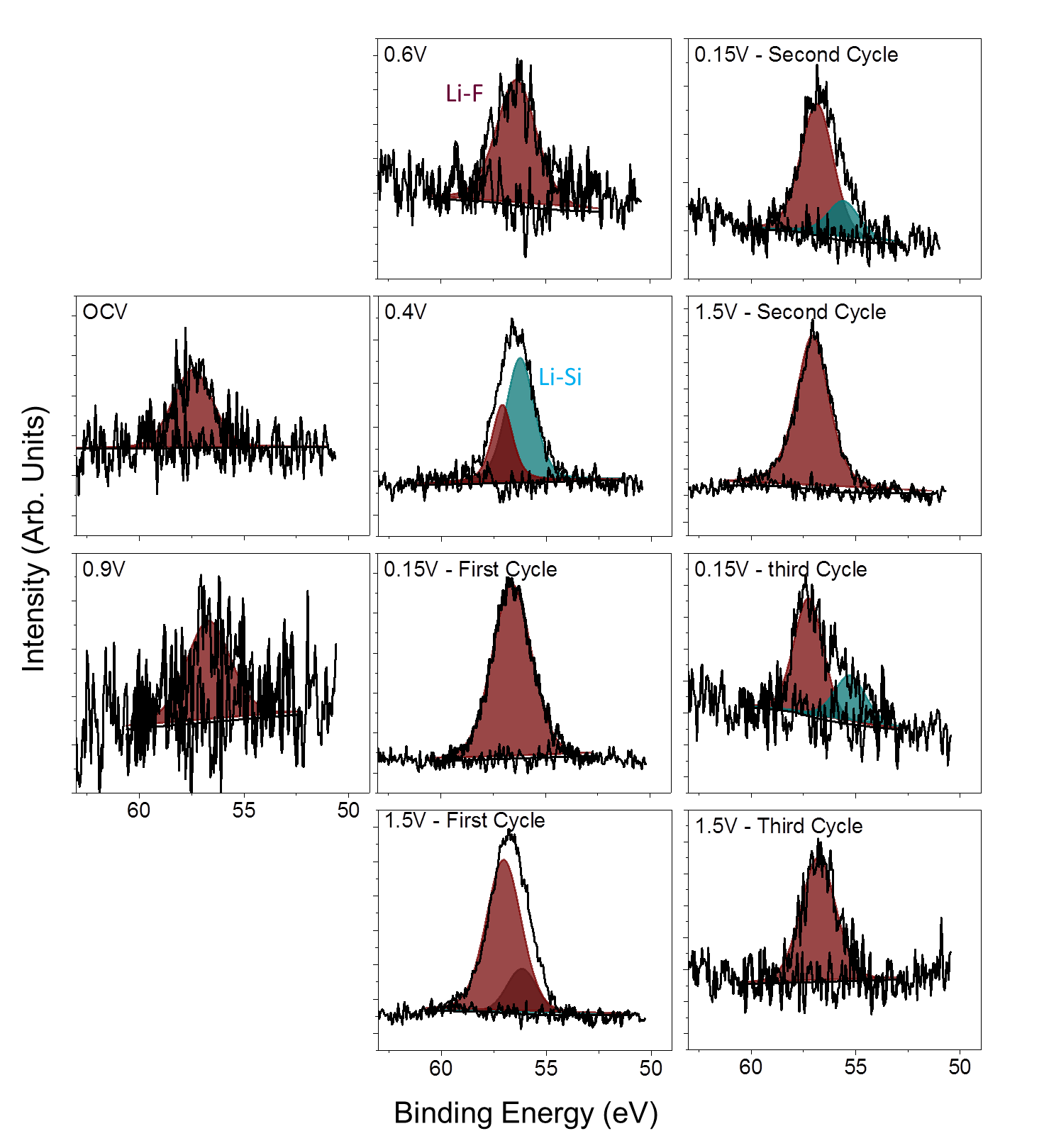


Figure 4. Measured and predicted Si thicknesses as a function of state-of-charge.


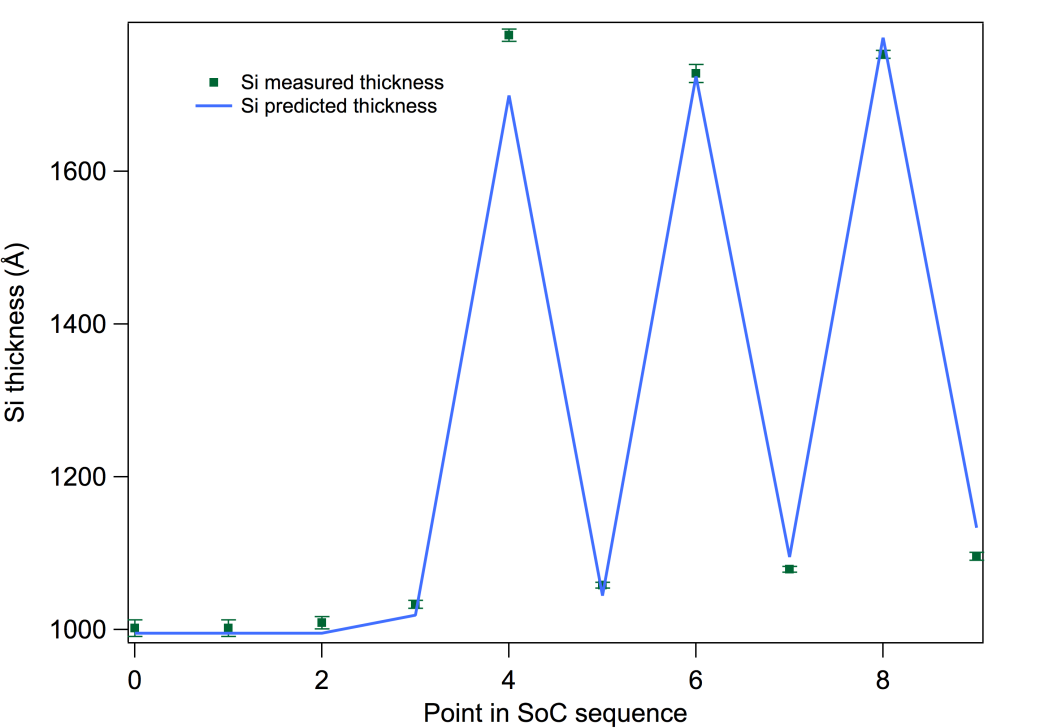


Figure S5. Comparison of SEI thickness and SLD for NR measurements with (left) and without FEC in the electrolyte.


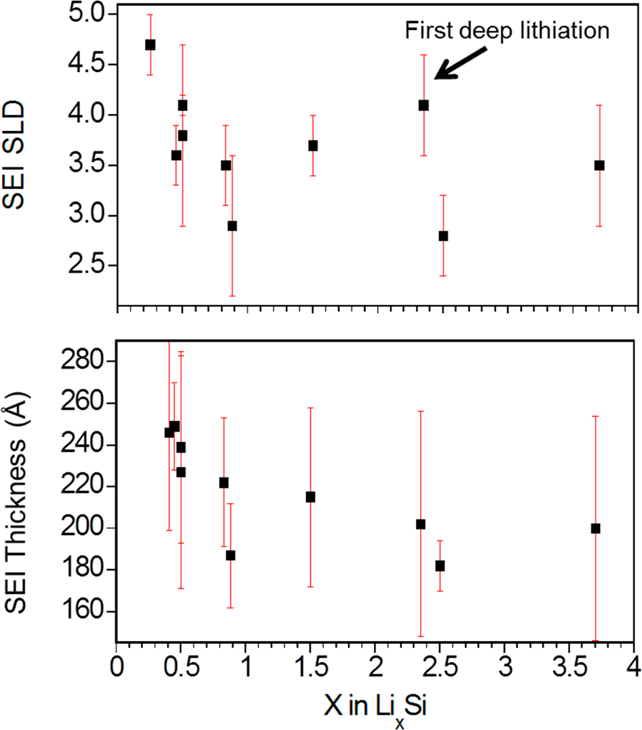


Figure S6. Summary of SEI thickness and SLD for FEC-free (top) and FEC containing (bottom) electrolytes.

Reproduced from ref [^42^](#_ENREF_42).


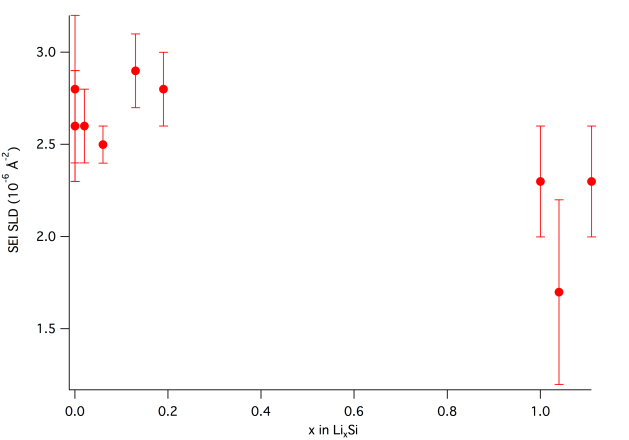

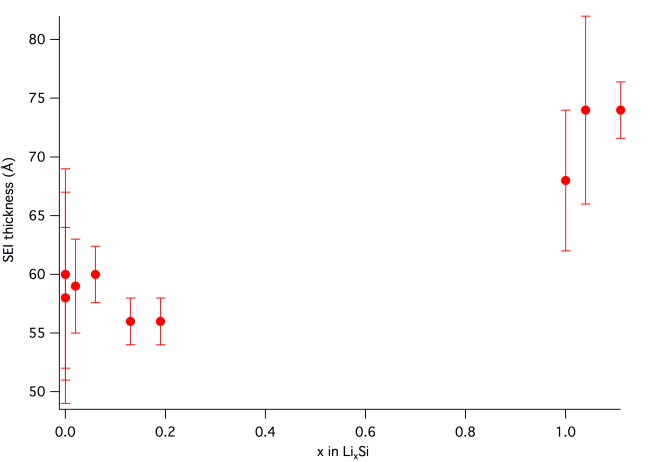


Table S1. Summary of XPS data measured for the cycled electrodes

| Sample | C1s | C1s | C1s | C1s | C1s | C1s | O1s | O1s | O1s | O1s | C-O-F-P-Li-Si |
| --- | --- | --- | --- | --- | --- | --- | --- | --- | --- | --- | --- |
|  | C-C, C-H | C-O | C=O | O-C=O, | -CO_3_ | C-F | Li-O | C=O | C-O, Si-O | C-O-C, P-O-F |  |
| Air | 285.49 | 286.78 |  |  | 289.28 |  |  | 531.09 | 532.74 | 534.20 | 46.1-25.0-0-0-0-28.9 |
|  | *70.1* | *20.2* |  |  | *9.7* |  |  | *10.4* | *66.7* | *22.9* |  |
| OCV | 285.05 |  | 287.05 |  | 289.08 |  |  |  | 533.19 |  | 17.4-19.3-24.8-4.4-8.0-26.2 |
|  | *71.1* |  | *24.6* |  | *4.3* |  |  |  | *100* |  |  |
| 0.9 V | 284.66 | 285.33 | 286.91 | 288.49 | 289.45 |  |  | 531.73 | 532.97 | 534.28 | 19.5-30.8-11.3-5.7-1.6-31.2 |
|  | *18.0* | *41.3* | *39.0* | *0.9* | *0.8* |  |  | *13.6* | *75.7* | *10.6* |  |
| 0.6 V | 284.64 | 285.59 | 286.66 | 287.82 | 289.52 |  |  | 531.56 | 533.02 | 534.28 | 42.6-20.8-14.3-1.7-9.9-10.7 |
|  | *16.7* | *59.3* | *11.9* | *6.8* | *5.3* |  |  | *19.8* | *61.9* | *18.3* |  |
| 0.4 V | 285.20 | 286.00 | 286.99 | 288.25 | 289.85 | 290.95 |  | 531.37 | 532.90 | 534.28 | 28.9-21.5-16.8-2.1-25.8-4.9 |
|  | *17.7* | *44.1* | *16.7* | *8.1* | *6.4* | *7.0* |  | *11.3* | *63.3* | *25.5* |  |
| 0.15 V 1^st^ | 284.94 | 286.46 | 287.90 | 289.04 | 290.02 | 291.06 | 530.68 | 532.39 | 533.32 | 534.50 | 32.4-25.3-8.2-0.9-32.8-0.3 |
|  | *11.8* | *56.8* | *12.8* | *1.8* | *3.9* | *12.8* | *9.6* | *13.4* | *57.6* | *19.4* |  |
| 1.5 V 1^st^ | 285.03 | 286.13 | 287.83 |  | 289.88 | 291.09 |  | 531.52 | 533.08 | 534.54 | 24.8-20.4-32.5-2.7-19.4-0.1 |
|  | *7.5* | *55.5* | *21.0* |  | *8.7* | *7.3* |  | *10.5* | *58.1* | *31.4* |  |
| 0.15 V 2^nd^ | 284.72 | 286.09 | 287.75 | 288.57 | 289.61 | 291.17 |  | 531.42 | 533.01 | 534.46 | 51.5-24.0-14.2-0.3-8.6-1.4 |
|  | *12.3* | *67.8* | *6.0* | *3.6* | *2.8* | *7.5* |  | *12.4* | *74.8* | *12.8* |  |
| 1.5 V 2^nd^ | 285.03 | 286.13 | 287.83 |  | 289.88 | 291.09 |  | 531.52 | 533.08 | 534.54 | 24.9-20.4-32.5-2.7-19.4-0.1 |
|  | *7.5* | *55.5* | *21.0* |  | *8.7* | *7.3* |  | *10.4* | *58.2* | *31.4* |  |
| 0.15 V 3^rd^ | 284.83 | 286.32 |  | 288.25 | 290.24 | 292.07 | 530.14 | 531.83 | 533.47 | 535.60 | 53.7-19.8-11.1-0.2-14.5-0.7 |
|  | *46.3* | *41.7* |  | *7.1* | *3.2* | *1.7* | *8.7* | *38.1* | *49.4* | *3.8* |  |
| 1.5 V 3^rd^ | 284.40 | 285.70 | 287.03 | 288.70 | 290.49 |  |  | 531.49 | 533.17 | 534.28 | 47.8-24.3-32.1-0.1-11.5-4.3 |
|  | *16.2* | *56.1* | *17.6* | *6.7* | *3.4* |  |  | *20.3* | *55.7* | *24.0* |  |

Table S1. Summary of XPS data as a function of state of charge.

Shifted relative to Si^o^ = 99 eV; Incommensurate charging of insulating inorganic LiF species.

Table S1. Summary of XPS data as a function of state of charge.

| Sample | F1s | F1s | F1s | P2p | P2p | P2p | Li1s | Li1s | Li1s | Si2p | Si2p | Si2p | Si2p |
| --- | --- | --- | --- | --- | --- | --- | --- | --- | --- | --- | --- | --- | --- |
|  | LiF | P-O-F | P-F | P-O | P-O-F | P-F | Li-Si | LiF | Li-PF_6_ | Si^o^ | Si^2+^ | Si^4+^ | Si-F |
| Air |  |  |  |  |  |  |  |  |  | 99.02 | 101.00 | 103.01 |  |
|  |  |  |  |  |  |  |  |  |  | *64.2* | *28.3* | *7.5* |  |
| OCV |  | 687.58 | 689.98 |  |  | 140.24 |  |  | 57.43 | 99.09 | 101.11 | 103.22 | 104.40 |
|  |  | *94.1* | *5.9* |  |  | *100* |  |  | *100* | *77.7* | *4.9* | *6.3* | *11.0* |
| 0.9 V | 686.92 | 688.21 | 689.69 | 135.03 |  | 140.16 |  | 56.64 |  | 99.03 | 102.82 | 103.93 |  |
|  | *73.7* | *12.4* | *13.9* | *59.5* |  | *40.5* |  | *100* |  | *82.5* | *6.1* | *11.4* |  |
| 0.6 V | 686.19 | 688.19 | 689.69 | 135.12 |  | 140.06 |  | 56.30 |  | 99.06 | 102.82 | 103.83 |  |
|  | *75.5* | *19.7* | *4.8* | *74.5* |  | *25.5* |  | *100* |  | *74.9* | *12.6* | *12.5* |  |
| 0.4 V | 686.37 | 688.46 | 689.69 | 134.74 |  |  | 56.22 |  | 56.93 | 99.11 | 102.94 |  |  |
|  | *87.1* | *9.9* | *3.1* | *100* |  |  | *56.0* |  | *44.0* | *66.7* | *33.3* |  |  |
| 0.15 V 1^st^ | 686.94 |  | 689.34 | 134.98 |  | 139.06 |  | 56.62 |  | 99.30 | 101.37 | 103.05 |  |
|  | *71.8* |  | *28.2* | *63.5* |  | *36.5* |  | *100* |  | *3.5* | *14.9* | *81.6* |  |
| 1.5 V 1^st^ | 686.59 | 688.44 | 689.44 | 135.12 | 137.38 |  |  | 56.96 |  | 98.99 | 102.63 | 103.98 |  |
|  | *80.7* | *7.5* | *11.8* | *55.2* | *44.8* |  |  | *100* |  | *69.3* | *15.4* | *15.3* |  |
| 0.15 V 2^nd^ | 686.74 | 688.88 | 689.86 | 134.77 | 138.06 |  | 55.60 |  | 56.87 | 99.36 | 100.71 | 103.47 |  |
|  | *79.2* | *10.8* | *9.9* | *92.5* | *7.5* |  | *23.6* |  | *76.4* | *1.0* | *1.0* | *98.0* |  |
| 1.5 V 2^nd^ | 686.56 | 688.44 | 689.44 | 135.15 | 137.38 |  |  | 56.86 |  | 98.99 | 102.63 | 103.98 |  |
|  | *80.7* | *7.5* | *11.8* | *55.2* | *44.8* |  |  | *100* |  | *69.3* | *15.4* | *15.3* |  |
| 0.15 V 3^rd^ | 684.77 | 687.17 | 689.34 | 135.45 |  |  | 55.29 |  | 57.26 | 99.24 | 101.67 | 103.40 |  |
|  | *7.7* | *80.0* | *12.3* | *100* |  |  | *32.7* |  | *67.3* | *20.0* | *39.0* | *41.0* |  |
| 1.5 V 3^rd^ | 686.47 | 688.80 |  | 134.67 |  |  |  | 56.72 |  | 99.15 |  | 103.42 |  |
|  | *92.3* | *7.7* |  | *100* |  |  |  | *100* |  | *28.5* |  | *71.5* |  |

Shifted relative to Si^o^ = 99 eV; Incommensurate charging of insulating inorganic LiF species
